# The Book World of Medicine and Science

**Published:** 1896-07-25

**Authors:** 


### The Book World of Medicine and Science.

Physics for Students of Medicine. By Alfred Daniell, M.A. (London: Macmillan and Co. 4s. 6d.)

Although the medical student aspiring to the higher degrees in medicine has for many years past been expected to show a rudimentary knowledge of physics, until within the last few years no such demands have been made obligatory on the student who contented himself with any of the humble qualifications. In 1892 the General Medical Council decreed that from that time forward all examining bodies possessing powers to grant diplomas should satisfy themselves that candidates had gone through a course of physics, and that they possessed an elementary knowledge on the subject. Since this regulation has been in force, teachers at the various medical schools have found the greatest difficulty in finding suitable text-books for their pupils. Ganot's and Dr. Daniell's "Principles of Physics" were too exhaustive and elaborate, and none of the smaller works contained in themselves all the range of subjects suitable for the preliminary study of medical physics. Dr. Daniell, who, perhaps, more than anybody else in Edinburgh, was responsible for this change in the medical curriculum, has brought all his wide experience to bear upon the subject, and has managed to compress into some 450 pages everything in physics that is important or necessary for a medical student to know, and without which he cannot take a broad or scientific view of the pathological and physiological problem which daily comes under his notice in the laboratory and wards. Dr. Daniell rightly insists that the student must supplement his theoretical knowledge with practical work, and to this end the scheme of the book is largely devised. The style throughout is admirably lucid, without a word of irrelevant matter, and the diagrams are adequate. There is, however, no index—a decided blemish to what otherwise is a most excellent students' text-book.

A New Treatment of So-called Incurably Deaf People. By Julian J. Hovent, M.D. (Liege: Aug. Bernard.)

It is very difficult to define quackery so as at the same time to take in all the quacks and exclude all the honest men, and this difficulty is not lessened by the fact that some very honest people are so convinced of the virtues of certain methods which they practise that they advocate them in a manner which cannot be easily differentiated from the ways of quacks. Now we are quite ready to believe that there are cases of deafness which, although they have failed to recover even under well-directed treatment administered by skilled physicians, are yet after all benefited by the use of the compressed air bath. But that is hardly the thesis which Dr. Hovent sets himself to enunciate. It is not the virtues of the compressed air bath in general that are sung by him, but those of "my apparatus" at "my establishment" and of certain "modifications which give the air bath such a powerful action," the nature of which he does not describe because the technical details would be "too tedious." We need not say that a pamphlet on these lines is hardly according to English ideas of the ethically correct.

